# An Overview of the Anatomical Distribution of Tetrodotoxin in Animals

**DOI:** 10.3390/toxins14080576

**Published:** 2022-08-22

**Authors:** Daria I. Melnikova, Timur Yu. Magarlamov

**Affiliations:** A.V. Zhirmunsky National Scientific Center of Marine Biology, Far Eastern Branch, Russian Academy of Sciences, 690041 Vladivostok, Russia

**Keywords:** biotoxin, tetrodotoxin, TTX, TTX-bearing animals

## Abstract

Tetrodotoxin (TTX), a potent paralytic sodium channel blocker, is an intriguing marine toxin. Widely distributed in nature, TTX has attracted attention in various scientific fields, from biomedical studies to environmental safety concerns. Despite a long history of studies, many issues concerning the biosynthesis, origin, and spread of TTX in animals and ecosystems remain. This review aims to summarize the current knowledge on TTX circulation inside TTX-bearing animal bodies. We focus on the advances in TTX detection at the cellular and subcellular levels, providing an expanded picture of intra-organismal TTX migration mechanisms. We believe that this review will help address the gaps in the understanding of the biological function of TTX and facilitate the development of further studies involving TTX-bearing animals.

## 1. Introduction

Tetrodotoxin (TTX), one of the deadliest natural toxins, has attracted the interest of researchers from various fields for decades. This non-protein, weakly basic, heat-resistant low-molecular-weight toxin selectively blocks voltage-gated sodium channels along the muscle and nerve cells [1]. Due to the wide distribution of TTX in nature, its ecological and biological characteristics are relevant to date.

Since the isolation and purification of TTX from pufferfish ovaries in the early 1950s [2,3], the search for this toxin in different groups of animals has been ongoing. Most studies have focused on commercial animals and some highly toxic amphibians. However, the search for TTX sources led to the study of the prey and symbiotic microflora of TTX-bearers. Hence, TTX was found in many organisms in addition to commercial fish, including flatworms, nemerteans, annelids, planktonic chaetognaths [4], and bacteria from different taxonomic groups [5]. With progress in the field of TTX detection, the investigations of TTX bearers have improved. In the early works, whole-body and known toxic organ extracts were analyzed. Since the 1990s, studies of tissue and cellular TTX distribution began. Since the 2000s, researchers have actively implemented experimental approaches to study TTX accumulation and excretion in animals. Research progress in the TTX field over the past 20 years has led to the accumulation of a large amount of data, which, however, did not reveal the mechanisms underlying TTX circulation in ecosystems and the bodies of animals. High inter- and intraspecies variances in TTX content and variations in experimental designs and screening studies significantly complicate the search for common patterns in toxin accumulation and usage by animals.

The current review summarizes the data on the intra-organismal distribution of TTX in TTX-bearing animals; its accumulation during animal development; and its accumulation, depletion, and excretion in animals under experimental conditions. Special emphasis is placed on TTX localization at the cellular and subcellular levels.

## 2. Intra-Organismal Distribution of TTX in TTX-Bearing Animals

### 2.1. Actinopterygii

The first experiments that led to the discovery of the toxins in the ovaries of fish belonging to the order Tetraodontiformes were conducted from the end of the 19th century to the middle of the 20th century [4]. The isolation of TTX and elucidation of its chemical structure led to the first large-scale toxicity studies of pufferfish inhabiting Japan [6,7]. Species of the genus *Takifugu*, particularly *Takifugu pardalis*, *Takifugu flavipterus* (*Takifugu poecilonotus*), and *Takifugu alboplumbeus* (*Takifugu niphobles*), are highly toxic. In subsequent years, TTX was found among most genera of the family Tetraodontidae dwelling in different countries and regions. High levels of toxicity have been observed in species of the genera *Arothron*, *Amblyrhynchotes*, *Chelonodon*, *Dichotomyctere*, *Lagocephalus*, and *Sphoeroides* [8,9,10,11,12,13]. TTX is also found in the species *Yongeichthys criniger* (*Gobius criniger*) of the order Gobiiformes [14,15,16,17].

**Figure 1:**
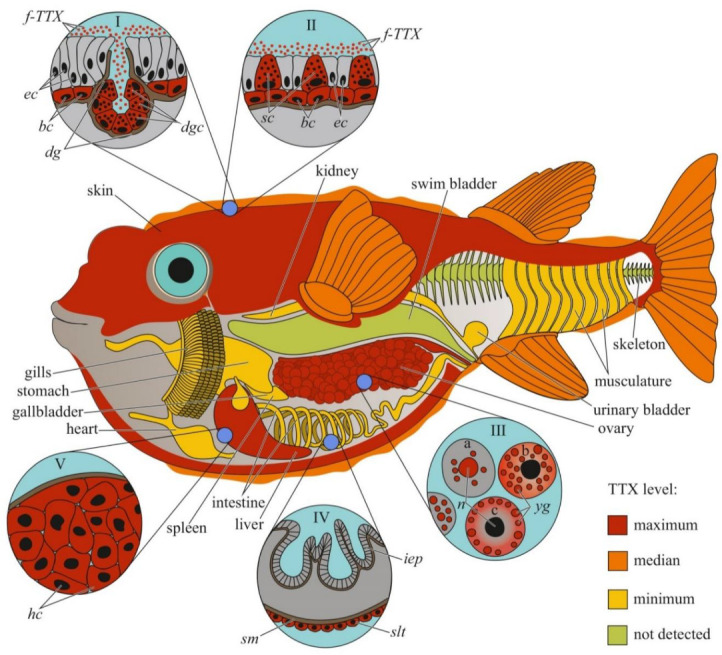
Schematic illustration of the levels and intra-organismal distribution of tetrodotoxin (TTX) in the adult pufferfish (family Tetraodontidae). Red color on the insets indicates TTX-positive cells. **I**—Skin with dermal gland (*dg*). **II**—Skin with singly scattered succiform cells (*sc*). **III**—Oocytes on different maturation stages: a—immature oocyte, b—newly mature oocyte, c—mature oocyte. **IV**—Intestinal epithelium (*iep*) and sac-like tissue (*slt*) outside the serous membrane (*sm*). **V**—Liver with hepatocytes (*hc*). **Abbreviations**: *ec*, epithelial cell; *bc*, basal cell; *dg*, dermal gland; *dgc*, dermal gland cell; *f-TTX*, free TTX; *sc*, succiform cell; *n*, nuclei; *yg*, yolk granules; *iep*, intestinal epithelium; *slt*, sac-like tissue; *sm*, serous membrane; *hc*, hepatocyte.

In previous studies, the authors reported either the general toxicity of the animal [18,19] or the toxicity of its ovaries [20]. Since the 1980s, the toxicity to individual organs and tissues has been studied. Researchers have mainly focused on the skin, muscles, intestines, gonads, and liver (or hepatopancreas, according to Sato et al. [21]); the fins, spleen, blood, and gills have been studied less extensively. Many pufferfish species have high concentrations of TTX in the ovaries, followed by the liver and/or skin; the remaining organs contain small amounts of TTX [7,8,11,22,23,24,25,26,27,28,29,30,31] (Figure 1). In *T. alboplumbeus*, the ventral skin is more toxic than the dorsal [25]. Comparative toxicity analysis of the fins and skin of *Takifugu vermicularis*, *Takifugu snyderi*, and *Takifugu porphyreus* showed that these tissues in some species had similar levels of toxicity [32]. In *Chelonodon patoca*, the highest toxicity is found in the skin; the muscles, liver, and ovaries of fish contain lower levels of TTX [27]. The brackish and freshwater species of the genera *Dichotomyctere* and *Pao* also have high concentrations of TTX in the skin compared to that in other organs [33,34] High concentrations of TTX can be found in the skin mucus of *Arothron meleagris* and *Sphoeroides lispus* [10]. In several species of the genus *Lagocephalus*, the gastrointestinal tract is the most toxic organ [13,35]. Artificially reared *T. rubripes* contain low concentrations of TTX accumulated in the ovaries and liver, similar to the observations in wild specimens [30,36]. Sato et al. [37] reported trace amounts of TTX in the guts of cultured *T. rubripes*. However, some studies reported the absence of toxins in cultured pufferfish [38,39].

The distribution of TTX within the body can vary depending on the habitat of the pufferfish. In *Lagocephalus lunaris* caught near Thailand [12] and the west coast of Japan [40], TTX is mostly localized in the gonads, while other organs, including the liver, skin, digestive tract, and muscles, contain low concentrations of the toxin. *L. lunaris*, which inhabits Cambodia, contains substantial concentrations of TTX in the liver, ovaries, and intestines, while the muscles, testes, and skin are less toxic [41]. The TTX content in different organs can also be affected by seasonal changes. In females of several species of the genus *Takifugu*, *Y. criniger*, and introduced species of the genera *Lagocephalus* and *Torquigener*, caught in the Mediterranean Sea, most of the TTX is contained in the ovaries in the autumn–winter period, during maturation, and in the liver and/or skin in the spring-summer period, after spawning [9,42,43,44,45,46,47,48,49]. Different data were obtained for males of different species of the genus *Takifugu*. *T. flavipterus* and *T. pardalis* do not show seasonal variations in TTX content [42,46]. In male as well as female *T. alboplumbeus*, an increase in the toxicity level in the pre-spawning and spawning periods was observed, when TTX was localized in the skin and liver [44]. *Lagocephalus* males contain substantial TTX concentrations in the testes only in the summer; other organs contain trace amounts of the toxin [45]. The concentration of TTX in the organs of *Torquigener flavimaculosus* males reaches the maximum level in winter and gradually decreases in autumn; in summer, only the levels in the testes and skin increase to winter values [47].

The cellular and intracellular localizations of TTX in pufferfish has been studied in highly toxic organs, including the skin, ovaries, and liver (Figure 1). Kodama et al. [50] detected TTX in secretions collected from the skin gland of *T. pardalis* and stated that the toxin was produced by the secretory cells of this gland. Later, the localization of TTX in the secretory cells of pufferfish skin was confirmed by immunohistochemistry using anti-TTX antibodies [21,27,51,52]. In *T. pardalis* [50], *T. vermicularis* [27], *T. flavipterus*, and *Canthigaster rivulata* [21], TTX-secreting cells are located in the glands or gland-like structures. In *C. patoca* [27], *Dichotomyctere ocellatus* (*Tetraodon steindachneri*) [51], and *Dichotomyctere nigroviridis* (*Tetraodon nigroviridis*) [52], TTX is located in the so-called succiform cells evenly scattered throughout the skin of the animal. Itoi et al. [53] showed that succiform cells of *T. alboplumbeus* males stained for TTX more intensely than the succiform cells of the females. TTX has also been found in the undifferentiated basal cells of pufferfish skin [43,51,52,53]. According to electron microscopic studies of *D. nigroviridis* skin, TTX in the basal cells is associated with membrane-bound granules (presumably lysosomes) [52]. The TTX absorption ability of the basal cells of the skin was demonstrated in experiments with intramuscular, intraperitoneal, and oral administration of toxins to non-toxic cultured pufferfish [54,55,56]. Interestingly, the intensity of staining for TTX in the skin cells depends on the dose of the injected toxin: low concentrations of injected TTX in *T. rubripes* only resulted in basal cell staining, while an increased dose stained the entire epidermis [56]. In vitro skin slices incubated in a TTX solution showed that TTX was transferred to the basal cells from connective tissue [57]. In addition to succiform and basal cells in the skin of *T. alboplumbeus*, TTX is found in mucous cells and the flat epithelial cell layer [53]. In the skin of *Y. criniger*, all epidermal cells, including filament-containing Malpighian [58], basal, and succiform cells, stained positively for TTX [43].

After studying the distribution of TTX in the cellular and subcellular liver fractions of *T. pardalis* and *Takifugu snyderi* (*Takifugu vermicularis snyderi*), Nagashima et al. [59] found that TTX was predominantly associated with the cytosolic fraction of the liver cells. Subsequently, immunocytochemistry with anti-TTX antibodies has been used to detect TTX in the cytoplasm of parenchymal hepatocytes in several species of *Takifugu* and *C. patoca* [21,27,53,55,57]. An in vitro experiment with the incubation of *T. rubripes* liver slices in a TTX solution showed that the toxin was transferred to the parenchymal hepatocytes from the pancreatic exocrine cells [57].

In the first study on the micro distribution of TTX in pufferfish ovaries, TTX was detected in the cytoplasm and membrane-limited yolk granules of pre-ovulated oocytes and the vitellin envelope of the ovulated oocytes [60]. In *T. vermicularis* ovaries, TTX is found in the yolk granules, vesicles, and nuclei of mature oocytes; immature oocytes do not contain the toxin [27]. In *C. patoca*, TTX is localised in the connective tissues and nuclei of some oocytes [27]. Itoi et al. found TTX in the oocyte nuclei of *T. alboplumbeus* [53]. Gao et al. [46] traced the localization of TTX during oocyte maturation in *T. pardalis*; in early maturation stages, TTX was localized in the nucleus and yolk granules, and late stages, in the cytoplasm and on the periphery of the cell.

Immunohistochemical studies have shown a weak TTX-positive signal in the sac-like tissues outside the serous membrane of the *T. flavipterus* intestine [21] and in the brain (optic tectum, cerebellum, and medulla oblongata), optic nerve, and olfactory epithelium of *T. rubripes* juveniles [55].

### 2.2. Amphibia

Newts are the most popular study animals among terrestrial TTX-bearers. In 1963, a toxin was isolated from the eggs of the newt *Taricha torosa* (*Triturus torosus*) [61], which was soon identified as TTX [62,63]. This discovery led to an array of toxicity studies on various members of the Salamandridae family. The first large-scale screening by Wakely et al. [64] showed the presence of TTX in several members of the genera *Taricha*, *Cynops*, and *Triturus* as well as in *Lissotriton vulgaris* (*Triturus vulgaris*), *Ichthyosaura alpestris* (*Triturus alpestris*), and *Notophthalmus viridescens*. To date, TTX has been found in 10 genera of Salamandridae residing in different regions of the world: *Taricha*, *Notophthalmus*, *Cynops*, *Pachytriton*, *Paramesotriton*, *Laotriton*, *Triturus*, *Lissotriton*, *Ichthyosaura*, and *Ambystoma* [64,65,66,67,68]. Differences in TTX content among individuals within populations have been reported for *Taricha granulosa* [69,70], *N. viridescens* [71], and *Cynops pyrrhogaster* [72].

**Figure 2:**
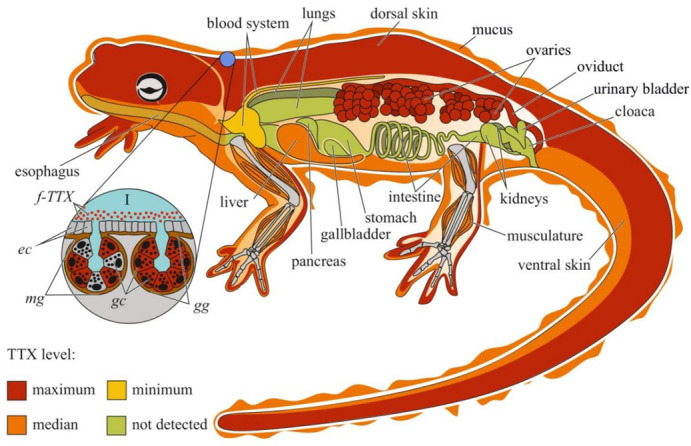
Schematic illustration of the levels and intra-organismal distribution of tetrodotoxin (TTX) in the adult newt (family Salamandridae). Red color on the insets indicates TTX-positive cells. **I**—Skin with mature granular (*gg*) and mixed (*mg*) glands. **Abbreviations**: *ec*, epithelial cell; *gc*, glandular cell; *gg*, mature granular gland; *mg*, mature mixed gland; *f-TTX*, free TTX.

Studies on individual organs and tissues of newts have revealed that TTX is usually localized in the skin and ovaries; other organs, including the muscles, blood, viscera, liver, and testes, contain low concentrations of the toxin [21,64,65,66,73,74] (Figure 2). In some newt species, high TTX concentrations have also been found in the liver [74] and muscles [75].

TTX was first visualized in the skin of *C. pyrrhogaster* by immunohistochemistry with anti-TTX antibodies [76]. In the juveniles of *C. pyrrhogaster*, TTX is contained in the glandular cells of immature dermal glands, in adult individuals, in the glandular cells of granular and mixed dermal glands [76]. Sato et al. [21] found TTX-positive cells in the dermal glands of only adult *C. pyrrhogaster*; TTX-positive structures were absent in the liver, intestines, testes, and ovaries. Mailho-Fontana et al. [77] detected TTX in the dermal glands and blood plasma of dermal capillaries of *T. granulosa*. The authors also revealed differences in the morphology of TTX-positive glandular cells and the structure and chemical composition of their secretions between individuals from TTX-bearing and non-toxic *T. granulosa* populations. In *N. viridescens*, the dermal glands stained the most intensively for TTX; in the connective tissues, liver, intestinal epithelium, ovaries, testes, and kidneys, the TTX-positive labelling was moderate [74]. Spicer et al. [78] hypothesized that similar to other newt species, *N. viridescens* might possess an increased number of granular glands in brightly pigmented spots on the dorsal skin, which might be associated with increased levels of TTX. Although no differences in TTX concentration were found between the red spots and neighboring skin without spots, juveniles with more dorsal spots possessed higher TTX levels. Uniform TTX-staining was observed in the tissues of nematodes, trematodes, and cestodes parasitizing the intestinal cavity of *N. viridescens* [79].

In the order Anura, TTX has been found in four genera: *Atelopus* (family Bufonidae) [80,81,82,83,84,85,86,87], *Colostethus* (family Dendrobatidae) [84], *Brachycephalus* (family Brachycephalidae) [88,89,90], and *Polypedates* (family Rhacophoridae) [91]. TTX in frogs was first reported by Kim et al. [80] in 1975. The authors detected TTX in the skin of *Atelopus varius* and *Atelopus chiriquiensis*; internal organs, muscles, and bones did not contain TTX. A similar TTX distribution has been observed in *Colostethus inguinalis* [84] and *Polypedates* sp. [91]. Later, TTX was also found in the liver and ovaries of the Anura representatives. In *Brachycephalus ephippium*, TTX is contained in the skin, liver, and ovaries [88,90], whereas in *Brachycephalus pernix*, only in the skin and liver [90]. However, the TTX concentration in the ovaries of *B. ephippium* was three times lower than that in the skin [90]. In contrast, the ovaries of *A. chiriquiensis* [81] and *Atelopus glyphus* [86] contained more toxins than the skin. Immunohistochemical studies of *Atelopus hoogmoedi* showed the presence of TTX in the hepatocytes, granular skin glands, and epithelial skin cells [87].

### 2.3. Mollusca

**Figure 3:**
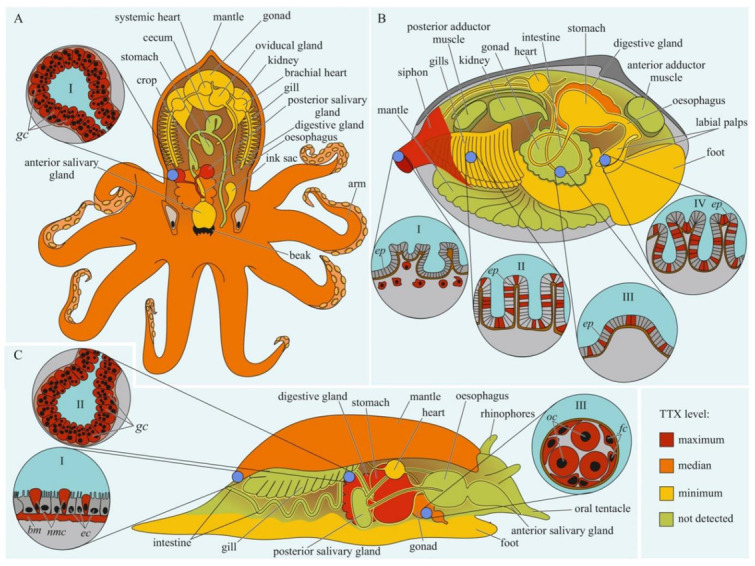
Schematic illustration of the levels and intra-organismal distribution of tetrodotoxin (TTX) in molluscs. Red color on the insets indicates TTX-positive cells. (**A**)—Blue-ringed octopus (genus *Hapalochlaena*). **I**—Posterior salivary gland with glandular cells (*gc*). (**B**)—Marine gastropod (genus *Pleurobranchaea*). **I**—The epidermis of the mantle. **II**—Digestive gland with glandular cells (*gc*). **III**—Oocytes (*oc*) surrounded by follicles (*fc*). (**C**)—Marine bivalve mollusc (genus *Paphies*). **I**—Siphon with TTX-positive cells located under the epithelium (*ep*). Gills (**II**), intestine (**III**), and labial palp (**IV**) showing epithelium (*ep*). **Abbreviations**: *gc*, glandular cells; *bm*, basement membrane; *ec*, epithelial cells; *nmc*, neutral mucin cells; *oc*, oocytes; *fc*, follicles; *ep*, epithelium.

The data on intra-organismal TTX distribution in octopuses, gastropods, and bivalves are summarized in Figure 3. TTX in molluscs was first discovered in 1978 when a toxin isolated from the posterior salivary glands of the octopus *Hapalochlaena maculosa* was identified [92]. Subsequently, TTX in *H. maculosa* was found not only in the salivary glands, but also in all body parts, including the arms, cephalothorax, and abdomen [93,94]. In *Hapalochlaena fasciata*, TTX has been found in the anterior and posterior salivary glands, arms, mantle, digestive gland, gonads, brachial heart, nephridia, gills, oviducal gland, and ink sac [95,96,97]. *Hapalochlaena lunulate* contains TTX in its salivary glands, gonads, mantle, arms, and ink [95,97,98]. However, the posterior salivary glands were the most toxic organs in both species. Immunofluorescence microscopy of the micro distribution of TTX in the tissues of *H. lunulate* and *H. fasciata* showed that TTX was concentrated in the cells lining the secretory tubules within the posterior salivary gland [97]. In the mantle and arms of *H. lunulate*, TTX was concentrated beneath the integumentary epidermis and in the channels of the circulatory system running through the dermis.

In 1981, a substance with a neuroparalytic effect and physicochemical properties similar to TTX was isolated from the digestive glands of the sea snails, *Charonia lampas* (*Charonia sauliae*) and *Babylonia japonica* [99,100]. Further studies revealed TTX in sea snails *Tutufa bufo* (*Tutufa lissostoma*) [101], *Buccinum undatum* [102], *Patella depressa*, and *Nucella lapillus* [103], as well as in some members of the families Naticidae [104,105], Trochidae [106,107], Nassariidae [108,109,110,111,112,113,114,115,116,117], and Olividae [118,119]. In most studies, TTX has been detected in the digestive glands. In the lined moon snail *Tanea lineata* (*Natica lineata*), the muscles are the most toxic part of the body, followed by the remaining parts, including the salivary gland, brain, and mouth organs; the digestive gland contains the least amount of TTX [120]. Hwang et al. [105] found that when seawater was released from the mantle cavity of *T. lineata*, TTX was secreted into the water. In another study, *Nassarius conoidalis* (*Niotha clathrata*) released TTX into the water in response to the first electric shock treatment; repeated stimulation did not cause toxin secretion [121].

TTX was also found in sea slugs *Pleurobranchaea maculata* [122], *Onchidella celtica*, and *Aplysia depilans* [103]. Wood et al. [123,124] reported high TTX concentrations in the stomachs of *P. maculata*, while the mantle and gonads of the mollusc were less toxic. A detailed study of the tissues and cells of *P. maculata* revealed the presence of TTX in the neutral mucin cells and basement membrane in the mantle, oocytes, and follicles in the gonads, and digestive gland [125]. TTX was found in *P. maculate* egg clutches [124,125].

The well known TTX-bearing bivalve mollusc is *Paphies australis* [126]. In *P. australis*, the highest level of TTX is contained in the siphon [127]. Immunohistochemical studies have shown that TTX was localized in the cells of the inner and outer epithelia of the siphon, in the inside cells of the epithelium of the intestine and rectum, and in the cytoplasm of some epithelial cells of the labial palps and gills [128]. Since 2008, TTX has been detected in many commercial bivalves living in European waters [102,103,119,127,129,130,131,132,133,134].

### 2.4. Echinodermata

The undigested body parts of starfish, found in the contents of the digestive glands of marine TTX-bearing gastropods, served as a starting point for the search for TTX in echinoderms. TTX has been found in several starfish species of the genus *Astropecten* [135,136,137,138] and in *Ophidiaster ophidianus* as well as in sea urchins *Echinus esculentus* [103] and *Fellaster zelandiae* (*Arachnoides zelandiae*) [139]. Lin et al. showed that the internal organs of *A. scoparius* had the highest toxicity, whereas the gonads and body wall were less toxic [106,140]. The tissue and cell distribution of TTX in echinoderms has not been studied.

### 2.5. Nemertea

For the first time, TTX was detected in two species of marine ribbon worms, *Lineus fuscoviridis*, and *Tubulanus punctatus* in 1988 [141], although pyridine compounds with a neurotoxic effect were previously found in extracts of *Amphiporus angulatus* [142]. In the nemertean *Cephalothrix simula* (in the article by *Cephalothrix linearis*), both the body and proboscis were found to be highly toxic, with the proboscis being the most toxic [143]. In addition, TTX was released into the mucus on the surface of the animal’s body during mechanical stimulation. Subsequently, TTX and its analogs have been found in representatives of the genera *Cephalothrix* [144,145,146,147,148,149,150,151], *Lineus* [147,149,150,152,153], *Ramphogordius* [149], *Riseriellus* [149], *Amphiporus* [149], *Yininemertes* [154], *Quasitetrastemma* [150], and *Collarenemertes* [150].

**Figure 4:**
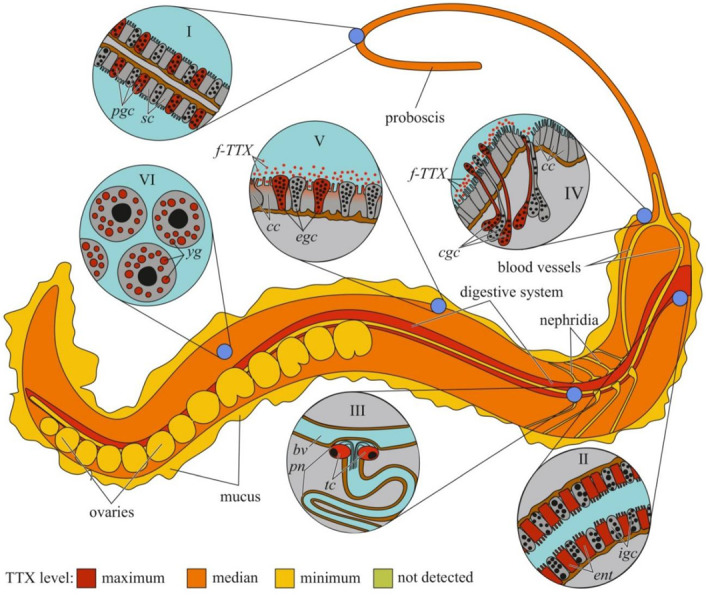
Schematic illustration of the levels and intra-organismal distribution of tetrodotoxin (TTX) in the marine ribbon worm (genus *Cephalothrix*). Red color on the insets indicates TTX-positive cells. **I**—Glandular epithelium of proboscis. **II**—Intestinal epithelium. **III**—Protonephridium (*pn*) associated with the blood vessel (*bv*). **IV**—Cephalic gland. **V**—Integument. **VI**—Oocytes. **Abbreviations**: *pgs*, proboscidial gland cell; *sc*, supportive cell; *ent*, enterocyte; *igs*, intestinal glandular cell; *bv*, blood vessel; *pn*, protonephridium; *tc*, terminal cell; *cc*, ciliary cell; *cgc*, cephalic gland cell; *f-TTX*, free TTX; *egc*, epidermal gland cells; *yg*, yolk granules.

The cellular and tissue localizations of TTX has been well studied in highly toxic nemerteans of the genus *Cephalothrix* [155,156] (Figure 4). In an early study on *C. simula* (*Cephalothrix* sp.), TTX was detected in the vesicles of the epidermal bacillary cells, basal lamina, granular cells of the proboscis epithelium, rhynchocoel epithelium, and vesicles of the intestinal epithelial cells located near the blood vessels and rhynchocoel [155]. The excretory system (protonephridia) and eggs also contain TTX. Vlasenko and Magarlamov found higher TTX content in the anterior region of the body of *C.* cf. *simula* than in the posterior one [151]. High TTX concentrations were detected in the intestine, body wall, proboscis, and mucus on the surface of the animal body. According to immunohistochemical studies, the main sites of TTX accumulation in *C*. cf. *simula* are the secretory cells of the integument, epidermal ciliary cells, mucous cells of the cephalic glands, glandular epithelium of the proboscis, enterocytes, and terminal cells of the protonephridia [156]. In the secretory and glandular cells, TTX is associated with secretory granules, in the ciliary cells, with microvilli, in the enterocytes, with phagosomes. In the protonephridial cells, TTX is located in the cytoplasm. In *Dushia atra* and *Micrura verrilli*, TTX was found in dermal glandular cells, intestinal epithelium, and outer proboscis epithelium, including pseudocnidae-containing cells [157]. In *Kulikovia alborastrata* (*Lineus alborostratus*), TTX was present in type-I subepidermal bacillary gland cells in the cutis and pseudocnidae-containing and mucoid gland cells in the proboscis [152]. Within glandular cells, TTX is associated with the nuclear envelope, the endoplasmic reticulum membrane, and secretory granules. Moreover, the glandular cells of the cutis in *K. alborostrata* released TTX-containing mucus in response to external stimuli [153].

### 2.6. Platyhelminthes

**Figure 5:**
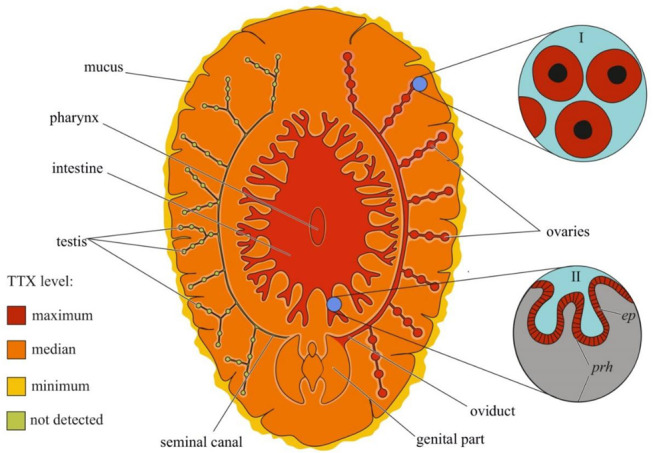
Schematic illustration of the levels and intra-organismal distribution of tetrodotoxin (TTX) in the marine flatworm (order Polycladida). Red color on the insets indicates TTX-positive cells. **I**—Oocytes. **II**—Pharynx showing epithelial cells (*ep*) and parenchyma (*prh*).

Although the toxicity of flatworms was reported in early naturalistic works from the 18th and 19th centuries, the first scientific work noting the presence of neurotoxins in marine and terrestrial flatworms dates back to 1943 [158]. After 40 years, TTX was found in extracts of the marine flatworms *Planocera multitentaculata* [159,160] and *Planocera reticulata* (type A) [161,162]. Subsequent studies showed the presence of TTX in several marine [125,163,164] and terrestrial [165] flatworms. In *P. multitentaculata*, the most toxic organs are the oviducts filled with mature eggs and organs of the digestive (including the pharynx) and reproductive systems; a weak neurotoxic effect of the mucus enveloping the worm was observed [160,166]. High TTX concentrations have also been found in the pharynx and eggs of *Planocerid* sp. 1 [163]. Detailed immunohistochemical studies have detected TTX in the cytoplasm of the ova and epithelial cells of the pharynx of *Stylochoplana* sp. [167] and the ova of *P. reticulata* [155]. In the terrestrial flatworms *Bipalium adventitium* and *Bipalium kewense*, most of the TTX was contained in the head and eggs; the anterior and posterior parts of the body contained smaller amounts of the toxin [165]. Figure 5 summarizes the intra-organismal TTX distribution in marine flatworms.

### 2.7. Annelida

In 1986, TTX was found in the annelid *Pseudopotamilla occelata* [168]. In their review, Miyazawa and Noguchi [4] mentioned that TTX was present in *Lepidonotus helotypus*, *Halosydna brevisetosa*, *Hermenia acanthopeltis*, and *Harmothoe imbricata*.

### 2.8. Chaetognatha

In 1988, TTX was found in planktonic chaetognaths *Parasagitta elegans* [169]; the sodium-channel-blocking activity of extracts of chaetognaths of the families Eukrohniidae (genus *Eukrohnia*), Sagittidae (genus *Flaccisagitta*), and Spadellidae (genus *Spadella*) was also reported; however, physicochemical methods of TTX detection were not used. Interestingly, only fractions containing chaetognath heads were toxic; the bodies did not contain TTX.

### 2.9. Arthropoda

TTX is found in three classes of arthropods: Malacostraca, Merostomata, and Copepoda. Among Malacostraca, TTX is detected in several crab species of the family Xanthidae [168,170,171,172,173,174,175,176]. Xanthid crabs contain TTX throughout their body, but the most toxic organs differ among species. In *Lophozozymus pictor* [171] and *Demania reynaudi* [174], the cephalothorax and viscera were found to be more toxic, whereas, in *Atergatis floridus*, the viscera and appendages were more toxic than other organs [174]. Saito et al. showed that the high toxicity of *A. floridus* was associated with the muscles of the chelipeds, particularly the muscles of the palm and carpus [177]. In some specimens, the muscles of the walking legs, gills, and ovaries were found to be toxic. In *Demania cultripes*, the viscera are more toxic than the appendages [176].

Among Merostomata, TTX is found in the horseshoe crab *Carcinoscorpius rotundicauda* [178]. In *C. rotundicauda*, TTX was first detected in the eggs [178]. Subsequent studies showed the presence of TTX in almost all organs and tissues of crabs of this species; however, most of the toxins are located in the eggs, muscles, viscera, and hepatic caecum [179,180,181].

Ikeda et al. first revealed the presence of TTX in the ectoparasitic copepod *Caligus fugu* (*Pseudocaligus fugu*) that parasitizes the pufferfish *T. alboplumbeus* [179]. In the same year, TTX was detected in another parasite of *T. alboplumbeus*, *Taeniacanthus* sp. [180]. Anti-TTX antibody-based immunohistochemical studies have shown that TTX in *C. fugu* was accumulated in all tissues of the body, intestines, and appendages except for the epicuticle and reproductive system, including the ovaries, oviduct, uterus, and egg sacs [179]. TTX appears at an early stage of chalimus development, and at chalimus stage IV and in adult copepods, it is present in all tissues and organs, except for the reproductive system [181].

### 2.10. Concluding Remarks

Data on TTX localization in animals can help elucidate the biological and physiological significance of the toxin. Most studies have focused on the anatomical distribution of TTX in animals, and only a small percentage of studies have focused on the intratissue and intracellular localization of TTX. A common tendency for TTX accumulation in the skin, ovaries, and endodermal organs (digestive system and liver) can be traced among different taxonomic groups of animals. The cellular distribution of TTX in these organs has mostly been studied in pufferfish (Figure 1) and nemerteans (Figure 4). Data on the cellular localization of TTX in other TTX-bearing animals are either limited or absent. More studies implementing high-resolution microscopy, such as laser scanning microscopy and electron microscopic immunocytochemistry, are required to trace the cellular and subcellular TTX distribution. These studies allow significantly expand the understanding of the cellular mechanisms of TTX sorption and migration within the body of a TTX-bearing animal. However, molecular mechanisms involved in TTX retention are yet to be explored. Studying the resistance of toxic pufferfish and arthropods to TTX, TTX-binding molecules in these animals were supposed [25,182,183,184]. TTX-binding proteins and high-molecular weight substances were isolated from the plasma of some pufferfish species, shore crab, and toxic gastropods [185,186,187,188,189] Nevertheless, the manner in which these molecules bind and transport TTX remains unclear. Future studies of the transcription factors involved in the regulation of TTX transport and accumulation in TTX-bearing animals are needed.

## 3. TTX in Animal Development: From Egg Maturation to Hatched Larvae

### 3.1. Actinopterygii

TTX was first isolated from the ovaries of *T. rubripes* in 1950 [190]. The high toxicity of the ovaries and the absence or low concentrations of TTX in the testes of most marine pufferfish species [191] led to studies of TTX kinetics in the maturation period. Studies on the seasonal changes in the TTX distribution in the tissues of *T. alboplumbeus* and *T. flavipterus* showed that TTX content in the ovaries increased sharply during egg maturation, and the total TTX concentration in the body of animals of both sexes was higher during maturation/spawning than in other months [42,44]. The ovaries of *Lagocephalus sceleratus* are toxic throughout the year, whereas the testes are toxic only in spring and autumn [45]. In *T. flavimaculosus*, the gonads are toxic in all seasons, except summer, and females are more toxic than males [47].

Using an indirect competitive enzyme immunoassay, TTX concentrations were calculated in *T. alboplumbeus* oocytes before and after ovulation [60]. The ovulated oocytes contained half as much TTX as pre-ovulated oocytes. Detailed studies of TTX distribution in the ovaries during oocyte maturation and spawning have been conducted on *Y. criniger* [43] and *T. pardalis* [46]. TTX accumulation in the ovaries of *Y. criniger* starts at the yolk globule stage, accounting for 45% of the total toxin in the body [43]. During the spawning period, the total toxicity of the female increases by up to eight times, where 73% of TTX is present in the ovaries. At the end of spawning, the toxicity of the female remains the same, but the TTX is mostly contained in the skin, and the contribution of the ovaries does not exceed 2%. Similar results were obtained for *T. pardalis*: during egg maturation, most of the TTX was concentrated in the ovaries, and after spawning, TTX content in the ovaries showed a sharp decline [46]. TTX is localized in the oocyte nucleus in the perinucleolar phase, redistributed to the yolk vesicles and globules during maturation, and partially transferred to the egg membrane close to spawning [46]. A similar study on *T. vermicularis* revealed TTX in the nuclei of oocytes in the perinucleolar phase, but in the yolk vesicles and granules at the final stage of yolk formation [27]. In the same study, the ovaries of *C. patoca* contained TTX only in the connective tissue and nuclei of some oocytes during the perinucleolar phase. In mature females of *T. alboplumbeus*, intense immunoreactivity against TTX in the oocyte nuclei was observed in both the perinucleolar and during yolk formation phases [53,60] whereas, in *T. flavipterus*, the oocytes stained intensively for TTX in the perinucleolar phase and weakly during maturation [21].

In 1998, Matsumura investigated TTX accumulation during the early development of *T. alboplumbeus* from fertilized eggs to hatched larvae [192]. After fertilization, TTX concentration in the developing embryos continuously increased. Immunohistochemical studies of *T. rubripes* and *T. alboplumbeus* larvae with anti-TTX antibodies showed even distribution of TTX over the body surface; no specific reaction was observed in internal organs [193,194]. Predation experiments with fertilised eggs and larvae of the above-mentioned pufferfish species and juveniles of different fish species showed that, despite low TTX content in eggs and larvae, predators promptly spat out the swallowed prey [193,194]. Nagashima et al. [195] traced the dynamics of TTX content during the development of *T. rubripes* for up to 98 days after hatching. The concentration of TTX per gram of body weight decreased during the growth and development of the animal, while the total content of the toxin increased.

### 3.2. Amphibia

TTX accumulation during the ontogeny of amphibians has mostly been studied in the Salamandridae family. The presence of a substance with a neuroparalytic effect in the embryos and eggs of newts was discussed as early as the 1930s [196,197,198]. While studying the growth of the eyes in newts, an accidental discovery was made: the transplantation of the eye vesicles of *T. torosa* embryos into *Ambystoma tigrinum* embryos led to temporary paralysis of the latter [197]. Parabiosis experiments with the embryos of two species of newts showed that the paralytic effect of the eggs decreased when the *T. torosus* yolk was eliminated and the effect completely disappeared when self-feeding juveniles formed. The authors also reported the paralytic effect of the extracts of the embryos, larvae, eggs, and blood of *T. torosus* females. The genus *Taricha* remains the most widely studied genus with respect to TTX accumulation during embryonic development. TTX has been found in the ovaries, testes (trace amounts), eggs, and embryos of *T. torosa* [64,65,199]; eggs of *T. granulosa* [65,200,201,202,203]; and *Taricha rivularis* [65]. TTX has also been found in the ovaries of *Cynops ensicauda* [66], egg clutches of *Cynops orientalis* [204], and eggs of *Paramesotriton hongkongensis* and *N. viridescens* [65]. Hanifin et al. [202] noted a positive correlation between the TTX contents in the dorsal skin and the egg clutches of *T. granulosa*. TTX concentrations in egg clutches differ between specimens [202], but clutches produced by the same female at different times contained eggs with similar levels of toxicity [200]. Moreover, TTX in the egg clutches of *T. granulosa* is unevenly distributed, and the amount of TTX decreases from the beginning to the end of the clutch [201]. 

The total amount of TTX in *T. granulosa* eggs does not significantly change during embryonic development, and the toxin is mostly concentrated in the embryo and not in the jelly coat [201]. Gall et al. analyzed the TTX content at different developmental stages of the *T. granulosa* larvae and evaluated their palatability to the predatory dragonfly nymphs *Anax junius* [203]. TTX concentration is the highest in newly hatched newt larvae, declines at 4 weeks of age, and remains relatively constant until the end of development (28 weeks). The palatability of newts was correlated with toxicity, given that older larvae and juveniles were preyed on more frequently than the new hatchlings [203]. Another study suggested that TTX secreted by newt skin could act as a deterrent signal, allowing larvae to avoid cannibalism from adults [205]. Behavioural experiments showed that adult *T. torosa* skin secretions or pure TTX triggered the avoidance of predation of the larvae. Similar behavioral responses were mostly observed in 3–5-week-old larvae and disappeared by the seventh week of development. Based on electrophysiological recordings and the lack of response to TTX in *T. torosa* larvae with a blocked nasal cavity, the authors claimed that the olfactory epithelium of the larvae possessed TTX-sensitive cells. Sato et al. [21] found TTX-positive immunoreaction in the pharynx and stomach of *C. pyrrhogaster* hatched larvae. In *C. pyrrhogaster*, artificially grown from eggs, TTX was detected only up to 22 weeks after hatching; older newts (from 36 to 70 weeks) did not possess TTX [206]; however, wild newts of the same age contained TTX. In a similar study involving *C. orientalis*, TTX was detected in adult wild newts and their egg clutches, but not in artificially reared larvae [204]. 

### 3.3. Mollusca

TTX has been found in the eggs, egg sacs, and larvae of the gastropod *P. maculata* [122,123,125,139,167] and blue-ringed octopuses [96,207,208,209]. An early study revealed low TTX concentrations in the egg sacs and 2-week-old larvae of *P. maculata* [122]. Furthermore, Wood et al. showed that the egg-laying season of *P. maculata* corresponded with the seasonal peaks of TTX concentrations (June–August) [124]. In another study, the authors revealed a correlation between TTX concentration in first-laid egg masses and adults of *P. maculata*; TTX concentration on a per wet weight basis in the egg masses released at the beginning of spawning was always higher than that in the spawning individuals [123]. In the egg sacs of *P. maculata*, TTX was localised in the eggs rather than in the surrounding matrix [167].

After the identification of TTX as the main component of the venom of *H. maculosa* [92], a TTX-like substance was isolated from the eggs of the octopus [207]. Williams et al. traced TTX concentrations at different developmental stages of the octopus *H. lunulata*, from undifferentiated eggs to hatched paralarvae [96,208]. TTX was present at all studied stages, increasing in concentration from the last stages of embryonic development (2–3 weeks after egg deposition) to the hatching of the formed paralarva (4 weeks after egg deposition). No correlation was observed between maternal TTX levels and larval developmental stage. TTX has also been found in the paralarvae of *H. fasciata* [209].

### 3.4. Others

High TTX concentrations, comparable to those in adults, have been found in the eggs and larvae of the marine flatworm *P. multitentaculata* [16,160,166,210,211,212] and eggs of *Stylochoplana* sp. [125], *Planocerid* sp. [163], and the terrestrial flatworm *B. adventitium* [165]. In *P. multitentaculata* larvae, TTX was found to be distributed over the body surface [212]. In *P. reticulate* eggs, the TTX concentration was not calculated, but the toxin was detected using immunohistochemistry with monoclonal anti-TTX antibodies [155]. In two immunocytochemical studies on marine ribbon worms, TTX was found in the cytoplasm of *C. simula* [155] and *C.* cf. *simula* eggs [156]. TTX has also been found in the eggs of the horseshoe crab *C. rotundicauda* [178,213,214,215,216,217] and the crab *A. floridus* [177]. However, the fate of TTX during embryogenesis in these animals remains unknown.

The pattern of TTX content in larval development has been described for the ectoparasitic copepod *C. fugu*: TTX is absent in planktonic larvae; at chalimus stage I and during subsequent feeding on the mucus of the host body, TTX appears in all tissues of the larva except the cuticle, gut, and some muscles; at chalimus stage IV and in adult animals, TTX is retained in the whole body except the reproductive system [181].

### 3.5. Concluding Remarks

Studies of TTX dynamics during individual development of TTX-bearing animals aim to clarify the role of the toxin in the offspring survival. Numerous studies showing high levels of TTX in the ovaries and eggs of TTX-bearing animals during maturation/spawning season suggest the participation of the maternal toxin in a fate of offspring. Predation and behavioral experiments with newts and pufferfish showed that TTX might functioning as a chemical defense against predators and, in some cases, cannibalism by older individuals. These studies revealed a positive correlation between the survival rate and TTX content in larvae or juveniles of TTX-bearing species. However, immunohistochemical studies did not reveal specialized cellular or subcellular structures allowing embryos or larvae of TTX-bearers retain TTX, indicating that the maternal toxin accumulated in the yolk during eggs maturation could be lost in the course of individual development. TTX dynamics studies show that concentration of the toxin in pufferfish and newts remains stable or increases during embryogenesis, and sharply decreases after hatching. The resorption of yolk during larval development can explain a rapid decrease in TTX level, but mechanisms retaining the toxin remain unclear. In this context, investigations of molecular basis of retention and maturation-dependent transportation of TTX are required. On the other hand, TTX appearance and role in the individual development of other TTX-bearing animals remains to be elucidated.

## 4. TTX Accumulation and Depletion Studies

The aim of TTX accumulation and depletion studies is to unveil the sources of the toxin. These studies usually involve oral or injectional administration of TTX to non-toxic lab-reared TTX-bearing animals (Table 1).

### 4.1. Actinopterygii

The first feeding experiment with non-toxic cultured pufferfish was conducted in 1981 [218]. Toxic pufferfish ovaries, methanol extract, and crystalline TTX were used as sources of TTX for non-toxic *T. rubripes*. The liver, skin, muscles, gonads, spleen, kidneys, and gallbladder of fish fed ovaries became toxic on the fifth day of the experiment, and by the 20th day, the toxicity gradually increased. In the fish of the methanol extract-feeding group, organ toxicity was lower than that in the other groups, but they became toxic by the end of the experiment. Interestingly, the fish fed crystalline TTX remained nontoxic. However, *T. alboplumbeus* juveniles fed crystalline TTX acquired toxicity and retained 30% of the administered toxin for 5 months [232]. TTX was found in the liver, skin, and ovaries of animals. In the work of Honda et al. *T. rubripes* of different ages accumulated TTX by ingesting both pufferfish tissues and their extracts and purified TTX [220]. The accumulated TTX was retained in pufferfish tissues for at least 45 days after the TTX-containing diet was completed. *T. alboplumbeus* juveniles fed highly toxic pufferfish livers accumulated up to 70% of the administered dose of the toxins [233]. The pattern of accumulation of TTX and its analog in the liver, skin, and intestines varied significantly. In the liver, the concentration of TTX accumulated during 30 days of the TTX-containing diet gradually decreased over the next 210 days of diet without TTX, whereas the content of 4,9-anhydro-TTX remained stable throughout the experimental period. In the skin, the contents of both toxins on the 30th day of the experiment were low and gradually increased toward the end of the observation period. In the intestine, TTX and 4,9-anhydroTTX were maintained at the same level throughout the experiment. Moreover, the selective pattern of TTX or paralytic shellfish toxins accumulation from the diet of pufferfish species naturally bearing one or the other toxin was revealed [235]. When *T. pardalis* was administered TTX and decarbamoylsaxitoxin (dsSTX), TTX accumulated in the liver, ovaries, and skin of the animal, and only trace amounts of dsSTX were detected in the intestine. In a similar experiment with TTX and STX, STX had accumulated in the ovaries and skin of the freshwater pufferfish *Pao suvattii*.

Intraperitoneal injection of tritiated TTX into *T. rubripes* lead to TTX accumulation in most tissues within an hour, with the highest level in the ventral skin [224]. The TTX levels in the liver and muscles were significantly low. On the sixth day of the experiment, TTX radioactivity levels decreased in most tissues, except for the skin and gallbladder. After intramuscular injection of TTX, 4-*epi*TTX, and 4,9-anhydro-TTX, *T. alboplumbeus* juveniles retained 34–40% of the toxins on day 16 after administration [234]. *T. rubripes* juveniles intramuscularly injected with pure TTX or toxic ovary extract retained approximately 60% of the injected toxin after 1–4 h [54]. After 8–12 h, TTX concentration decreased and again increased to 60–80% after 24–168 h. Low TTX concentrations were retained in the liver, whereas most of the toxin accumulated in the skin and was localized in the basal cells of the epidermal layer. Several subsequent studies have shown that in the first hours after intramuscular injection of TTX to pufferfish, most of TTX accumulated in the liver, and then a substantial part of the toxin has been transported to the skin and/or ovaries [227,230,231]. As the concentration of TTX in the liver decreased, the toxin appeared in the gallbladder, indicating excretion by the bile ducts [227]. Tatsuno et al. showed that TTX concentration in the skin and liver of *T. rubripes* increased with an increase in the dose of intramuscularly administered toxin, but the TTX accumulation ratio (ratio of accumulated TTX in each tissue to the administered dose) differed significantly between the tissues [56]. In the liver, the TTX accumulation ratio did not depend on the administered dose, whereas in the skin, it decreased with an increase in the dose.

Tatsuno et al. [221] found that the TTX distribution profile in *T. rubripes* in the first 24 h after toxin administration was dependent on the developmental stage of the liver. Individuals with a high hepatosomatic index retained 84% of the administered TTX, which mostly accumulated in the liver. Younger fish with an undeveloped liver retained only 31% of the administered TTX, mostly in their skin. In 3-month-old *T. obscurus* juveniles, up to 73% of accumulated TTX was localized in the skin, whereas less than 20% of the toxin occurred in the liver [236]. In vitro experiments with liver tissue slices incubated in TTX solution demonstrated that, unlike other fish, the pufferfish liver was able to accumulate TTX [118,241,242,243]. However, no difference was observed in TTX uptake between the livers of juvenile and adult pufferfish [244]. A recent study examined the TTX uptake ability of the liver, skin, and intestines of young (8 months old) and adult (20 months old) *T. rubripes* individuals [57]. The TTX uptake ability of the liver and intestines did not differ significantly between the two age groups, but the skin of young fish accumulated almost twice as much TTX as the skin of the adult.

In most experiments, other than pure TTX, toxic pufferfish organs or their extracts were used, and in a few studies, other TTX-bearing organisms were used as a source of the toxin (Table 1). In an early experiment, cultured *T. rubripes* (10 individuals) and *T. alboplumbeus* (three individuals) were fed a diet enriched with the TTX-producing bacterium *Shewanella putrefaciens* [219]. However, after 30 days of feeding, only one *T. rubripes* individual contained TTX in its liver. The successful toxification of *T. alboplumbeus* was achieved in a predation experiment with the flatworm *P. multitentaculata* [210]. TTX was detected in the liver, skin, and intestines of the pufferfish. Interestingly, the fish fed a weakly toxic planocerid ingested almost all the TTX, whereas the fish fed a highly toxic worm ingested only approximately 20% of the toxin. Zhang et al. did not reveal a direct effect of TTX concentration in *Nassarius semiplicata* on toxin accumulation in *T. obscurus* fed molluscs [236]. The accumulation ratio of TTX ranged from 35.76% in pufferfish fed moderately toxic molluscs to 40.20% when fed low-toxicity molluscs.

### 4.2. Amphibia

Studying the distribution of TTX in the tissues and organs of several species of newts, Wakely et al. found similar TTX levels in the body of *T. torosa* kept in the laboratory for 1 year and newly caught animals [64]. Several subsequent studies have shown that newts can retain TTX for a long period in captivity. Thus, the weight and general toxicity of *T. granulosa* kept in captivity for 167 days decreased, but the toxicity per gram of body weight remained almost the same [245]. In a study by Hanifin et al., TTX levels in the skin of *T. granulosa* not only remained unchanged, but also increased by an average of 20% after a year of maintenance in laboratory [73]. Wild-caught *T. granulosa* continued to produce eggs containing substantial amounts of TTX for 3 years in captivity [200]. In lab-reared *T. granulosa* juveniles kept on a non-toxic diet for 3 years, slow TTX accumulation in the skin during the first 2 years was observed [246]. In the third year, there was a sharp increase in toxicity. However, the authors noted that wild-caught *T. granulosa* juveniles were more toxic. Opposite results were obtained by Yotsu-Yamashita et al. in their experiments with *N. viridescens* [71]. Six years in captivity led to a complete loss of TTX, and only trace amounts of the toxin were detected in newts after 3 years. Long-term TTX retention under experimental conditions has also been observed in *Atelopus* frogs. *Atelopus oxyrhynchus* and *Atelopus subornatus* contained TTX, 4-*epi*TTX, and 4,9-anhydroTTX after more than 3 years in captivity [83,85]. At the same time, *A. varius* reared in the laboratory for more than 2 years did not contain TTX [247].

Juveniles of *C. pyrrhogaster* fed toxic pufferfish ovary extracts accumulated substantial amounts of TTX and retained them for a week, while the levels of the accumulated toxin differed between populations [72]. Recent studies on *C. pyrrhogaster* fed TTX and/or its putative biosynthetic intermediates showed that ingested compounds accumulated in the animals, but their conversion to other TTX analogs did not occur [21,237]. TTX has been detected in the tail, intestine, liver, skin, and ovaries of experimental animals [21]. Immunohistochemical studies of *C. pyrrhogaster* juveniles after feeding showed the presence of TTX in the epithelial cells of the intestine, mucoid skin cells, and ovaries; in the tail, TTX was localized in the mucoid skin cells and dermis [21].

### 4.3. Mollusca

Narita et al. conducted the first feeding experiment with the non-toxic gastropod Charonia sauliae [238]. The molluscs were fed TTX-bearing specimens of the starfish, A. polyacanthus. The digestive glands became toxic after a week of feeding, and the average TTX accumulation was 33%. An increase in the dose of TTX led to an increase in the total amount of accumulated toxin. When toxified molluscs were subsequently maintained on a TTX-free diet, no TTX metabolism and/or excretion from the body was observed. In a feeding experiment with P. maculata, TTX was detected in all the tissues within an hour of feeding [139]. The molluscs initially accumulated about 32% of the administered TTX, and the percentage of accumulated TTX gradually decreased to 9% on the 39th day of the experiment. High TTX concentrations were detected in the mantle of the molluscs. Biessy et al. conducted an interesting feeding experiment with P. australis using encapsulated TTX [240]. The bivalves fed agar-gelatine capsules with TTX bound to humic acid for 13 days actively accumulated toxins at concentrations exceeding the allowable norms in food products. However, the level of accumulated TTX did not exceed 0.5–1% of that contained in the food. When non-toxic marine snails Nassarius (Pliarcularia) globosus and Reticunassa festiva were fed toxic ovaries of T. vermicularis, the TTX accumulation ratios were 4% and 2%, respectively [239]. The non-toxic mussels Mytilus galloprovincialis were toxified after feeding on toxic *P. multitentaculata* larvae; TTX was detected only in the intestines of the animals [212]. Long-term maintenance of molluscs on a non-toxic diet after a week of feeding on planocerid larvae resulted in a sharp decrease in the TTX concentration.

In a study by McNabb et al. [122], toxic *P. maculata* specimens maintained in aquaria retained TTX for 26 d. In a large-scale study with *P. maculata* kept on a TTX-free diet for 126 days, the average TTX concentrations in the molluscs declined over time [123]. The depuration time differed among organs, with the fastest depuration observed in the heart and the slowest, in the gonads. The foot, mantle, and stomach were depleted of TTX at the same rate. Depuration of TTX during long-term maintenance without the toxin (150 days) has also been found in *P. australis* [248]. The siphons had the highest TTX concentrations during the entire observation period. The lowest concentrations of TTX and fast depuration were detected in the digestive glands, whereas after 21 days, only trace amounts of the toxin remained.

### 4.4. Non-TTX-Bearing Animals

An early study of the skin toxicity of *T. granulosa* revealed its activity against various vertebrates, including mammals of the orders Rodentia, Carnivora, and Eulipotyphla; birds of the order Passeriformes; reptiles of the order Squamata; and the newt itself [249]. As a result of forced or voluntary feeding on newt body parts or injection of the newt skin extract, all tested animals showed symptoms characteristic of TTX poisoning. However, snakes of the genus *Thamnophis* were resistant to TTX. Williams et al. [250] suggested that snakes that fed on highly toxic newts had substantial amounts of TTX in their tissues. *Thamnophis sirtalis* that fed on toxic *T. granulosa* specimens for 5 weeks retained TTX in the liver for 7 weeks and in the kidneys for 3 weeks [250]. Moreover, TTX was detected in the liver of *T. sirtalis* one month after ingestion of only one newt. In an experiment of oral TTX administration to newly hatched *T. sirtalis*, the half-life of TTX excreted from the liver was 8 days [97]. The authors found that 99% of a single TTX dose in the experiment would be eliminated from the snake’s body within 61 days.

Gall et al. [251] examined the ability of aquatic macroinvertebrates sympatric with *T. granulosa* to consume toxic newt eggs. Among the animals tested, only caddisfly *Limnephilus flavastellus* larvae could consume substantial quantities of toxic eggs, without this affecting their further development. Another study involving wild-caught *L. flavastellus* larvae and lab-reared larvae additionally fed with *T. granulosa* eggs showed higher toxin levels in the latter [252]. Interestingly, wild-caught larvae kept on a TTX-free diet retained the toxin for up to 134 days, even during metamorphosis and adult stage.

### 4.5. Concluding Remarks

Despite numerous studies elucidating the ways of TTX accumulation in marine and terrestrial animals and ecosystems, the problem of TTX biogenesis remains unresolved. Additionally, if the generally accepted hypothesis of TTX origin in nature is its bacterial production, as a source of toxin in animals, symbiotic microflora (endogenous), diet (exogenous), or combination of both factors are considered. TTX production by symbiotic and free-living bacteria and problems concerning the contribution of the microbiome in the toxification of the animal have been widely discussed in other reviews [1,5,191,253] and are not considered in this work. Here, we discuss challenges facing authors in the interpretation of experimental studies revealing TTX origin in animals.

Most scientific evidence indicates different toxification patterns between terrestrial and marine animals. Experiments with the long-term maintenance of wild-caught TTX-bearing newts on a TTX-free diet revealed endogenous acquisition of the toxin. In the similar experiments with marine molluscs, rapid TTX depuration was observed. Difficulties occur comparing results of this type of studies even within amphibias. Thus, species of the genus *Taricha* and some *Atelopus* frogs kept in laboratory for a long period retain or even increase TTX level, while *N. viridescens* and *A. varius* lose the toxicity. The main problem facing researchers is the measurement of the initial TTX concentration in the experimental animal. The average concentration of TTX within a given population or the toxin content in a small patch of skin are usually used as a starting point for investigation. At the same time, TTX content can significantly vary both within population and on different skin parts of one animal. As a consequence, it is difficult to trace actual depuration rate of TTX.

Feeding experiments held on marine and terrestrial animals and results of observations clearly indicate that TTX-bearers can accumulate exogenous TTX, through the food web. Using different methods of TTX administration, researches traced TTX kinetics inside animal body. All of these studies are based on the unique experimental designs different in basic parameters, such as time of TTX administration, observation period, and even organs investigated. Disparate data, on the one hand, significantly expand the understanding of TTX migration inside animals, and on the other hand, do not provide the understanding of common patterns and exact mechanisms of TTX accumulation. Different approaches, including a unified design of experiments and studies of separate organs or tissues, could potentially improve the TTX kinetics understanding. For instance, an in vitro experiment with pufferfish liver tissue slices revealed the involvement of carrier-mediated transport system in TTX uptake [118]. Future researches elucidating the molecular aspects of TTX transport in TTX-bearing animals are needed.

## 5. Conclusions

**Figure 6:**
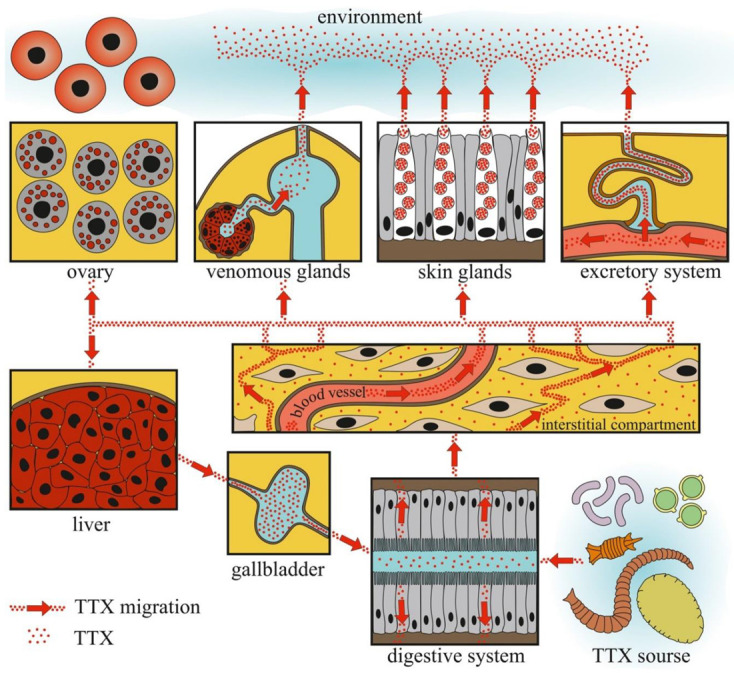
Schematic illustration of tetrodotoxin (TTX) migration in TTX-bearing animals.

Figure 6 summarizes the currently available data on the localization of TTX in the bodies of TTX-bearing animals in the natural environment and experimental conditions. 

The data obtained indicate that TTX can be absorbed into the body through the gastrointestinal tract. Cases of poisoning and feeding experiments showed that the migration rate of TTX from the intestinal cavity was quite high. The concentration of TTX detected in the intestinal extracts of TTX-bearing animals was minimal, indicating that TTX does not remain in the intestinal epithelium for a long time. High TTX concentrations in the intestines of the pufferfish species *Lagocephalus* and nemerteans may be related to the consumption of large quantities of TTX-bearing objects and/or slow migration of TTX through the intestines [13,35,151]. From the intestinal cavity, TTX is absorbed by phagocytic enterocytes [155,156].

Furthermore, TTX migration within the body depends on the development and features of the circulatory system. In animals with well-developed circulatory systems, most of the TTX enters the bloodstream and is distributed throughout the body. In most experiments with oral TTX administration to pufferfish, TTX was first detected in the liver and then in other organs and tissues (Table 1). In animals with a simple or absent circulatory system, TTX was mostly detected in organs localized near the anterior intestine. In nemerteans, the anterior parts of the body wall, proboscis, and intestines contained high levels of TTX [151]. In molluscs, the most toxic organs were the digestive and/or salivary glands adjacent to the esophagus and/or stomach (Figure 3, Figure 4 and Figure 5). Relatively high levels of TTX have also been found in organs that penetrate hemal vessels (gills and heart) and lacunae (mantle, leg, gonads, and siphon).

TTX-bearing animals kept on a TTX-free diet can retain TTX in the integument and excrete it with mucus and eggs for a long time. The data obtained suggest the presence of a reservoir for TTX in the animal body. The liver, which contains high concentrations of TTX, can be a toxin reservoir in TTX-bearing vertebrates. The liver, which obtains TTX through the blood, retains a large amount of the toxin and slowly releases the excess through the gallbladder into the gastrointestinal tract, where it is reabsorbed. A similar mechanism of TTX reabsorption has been suggested for the pufferfish *T. rubripes* [227]. Molluscs probably do not possess a TTX reservoir because the toxin quickly depurates under TTX-free conditions [123]. An experiment with electric stimulation and long-term maintenance in aquaria showed that, in nemerteans, the body wall could serve as a storage compartment for TTX [153].

The organs that release TTX in the environment include the gonads, excretory organs (kidneys or protonephridia), integument, and venom glands. In these organs, unidirectional transport of TTX can be assumed. TTX detection in the protonephridia of marine worms [158,159] and kidneys of pufferfish [218,224,227,228] indicates that the toxin is released through the excretory system. In the gonads, TTX is predominantly associated with egg-yolk granules and is released during yolk resorption during embryonic development [81,196,198,203]. In the glandular cells of the integument and venom glands, TTX was associated with secretory granules (Figure 1, Figure 2, Figure 3, Figure 4 and Figure 6). In the ribbon worm *K. alborostrata*, the migration of TTX through the organelles of protein synthesis (nuclear membrane, cisterns of the endoplasmic reticulum, and young glandular granules) to mature secretory granules and subsequent excretion into the environment was traced.

Other possible pathways for TTX entry into animals remain unclear. Based on the data obtained from nemerteans and pufferfish, TTX absorption through epithelial cells in the skin can be assumed. In *C*. cf. *simula*, the apical part of ciliated skin cells gradually stained for TTX: the distal part of the cell stained brightly, and the intensity gradually decreased toward the proximal part of the cell [156]. Small amounts of TTX have also been detected in the surface epithelial cells of *T. alboplumbeus* [53]. However, additional studies are needed to elucidate the mechanisms of TTX entry into the cells of the integumentary epithelium.

## Figures and Tables

**Table 1:** Summary of the experimental studies on tetrodotoxin (TTX) administration to non-toxic individuals of TTX-bearing animals.

Species	Method of TTX Admn.	TTX Source	Assayed Organs/Tissues	Most Toxic Organs	Time of TTX Admn.	Observation Period after TTX Admn.	Accumulated TTX (%)	Reference
*Takifugu rubripes*	Oral	CRY EXT TISS	Liver Skin Gonad Muscle Spleen Kidney GB	Liver Spleen GB	20 days	NA	NA	[218]
		BACT	Liver Skin Intestine	Liver	30 days	NA	NA	[219]
		TISS EXT CRY	Liver Skin Viscera	Liver Skin	60 days	45 days	Up to 30%	[220]
		EXT	Liver Skin Brain Olfactory Eye	NA	5 days	NA	NA	[55]
		CRY	Liver Skin Gonad Muscle GIT	Liver	One time	24 h	Up to 84%	[221]
		TISS	Liver Skin Intestine “Rest”	Liver Skin	9 days	NA	NA	[222]
		CRY	Liver Skin Muscle	NA	28 days	NA	NA	[223]
	Intraperitoneal injection	CRY	Liver Skin Muscle Intestine Spleen Kidney Heart Blood Brain Eye Bone Gill Mucus GB	Liver Skin Muscle intestine	One time	6 days	~35%	[224]
	Intramuscular injection	CRY TISS	Liver Skin Muscle Blood	Skin	One time	7 days	Up to 80%	[54]
		TISS	Liver Blood	NA	One time	12 h	68 ± 4%	[225]
		TISS	Liver Skin Gonad Muscle	Liver Gonad	One time	5 days	~70%	[226]
		CRY	Liver Skin Muscle Spleen Kidney Blood GB	Liver Skin	One time	24 h	~50%	[227]
		CRY	Liver Skin Muscle	Liver Skin	One time	24 h	Up to 40%	[56]
	Hepatic vein injection	TISS	Liver Skin Muscle Spleen Kidney Blood	Liver	One time	60 min	Up to 76%	[228]
	Hepatic portal vein injection	TISS	Liver Blood	NA	One time	300 min	84 ± 6%	[229]
	Hepatic vein injection						70 ± 9%	
	Gastrointestinal tract injection						49 ± 17%	
*Takifugu rubripes*/*Takifugu alboplumbeus* hybrid	Intramuscular injection	CRY	Liver Skin Gonad Muscle Blood	Skin Gonad	One time	3 days	Up to 65%	[230]
*Takifugu rubripes*/*Takifugu porphyreus* hybrid	Oral	CRY	Liver Skin Muscle Blood GIT	Liver Skin	One time	5 days	Up to 45%	[231]
	Intramuscular injection						Up to 74%	
*Takifugu alboplumbeus*	Oral	CRY	Liver Skin Gonad Muscle Bone Viscera	Liver Skin	30 days	170 days	~50%	[232]
		TISS	Liver Skin Gonad Muscle Intestine Bone Viscera	Liver Skin	30 days	240 days	~ 70%	[233]
		FWL	Body	NA	2 days	NA	NA	[210]
		FWA	Liver Skin Gonad Intestine “Rest”	Liver	2 days			
	Intramuscular injection	CRY	Body	NA	One time	16 days	Up to 35–40%	[234]
*Takifugu pardalis*	Oral	TISS	Liver Skin Gonad Muscle Intestine	Liver Skin gonad	One time	3 days	Up to 55%	[235]
*Takifugu obscurus*	Oral	MOL	Liver Skin Muscle Intestine Kidney Gills Blood GB AB	Liver Skin	28 days	67 days	Up to 40%	[236]
*Cynops pyrrhogaster*	Oral	EXT	Body	NA	9 days	6 days	NA	[72]
		NT-EXT	Body Viscera	Body	One time	28 days	~39%	[237]
		TISS	Liver Skin Gonad Intestine Tail	Tail	70 days	NA	NA	[21]
*Charonia lampas*	Oral	SF	DG Body	DG	7–28 days	40 days	~33%	[238]
*Pleurobranchaea maculata*	Oral	MOL-EXT	Gonad Stomach Mantle “Rest” Eggs	Mantle Stomach eggs	39 days	NA	~9%	[139]
*Nassarius globosus*	Oral	TISS	Muscle Viscera	Viscera	8 cycles: 1 day (TTX) + 4 days (no food)		<4%	[239]
*Reticunassa festiva*							<2%	
*Paphies australis*	Oral	CRY	DG + Siphon “Rest”	DG + Siphon	12 days	NA	0.5–1%	[240]
*Mytilus galloprovincialis*	Oral	FWL	Gonad Muscle Gill Mantle MG “Rest”	MG	1 day	28 days	NA	[212]

NA, not applicable; CRY, crystalline TTX; EXT, extract of pufferfish tissues (ovary or liver); TISS, puffer-fish toxic tissues (minced ovary or liver, eggs); BACT, bacterial cells; FWA, adult flatworm; FWL, flatworm larvae; MOL, mollusc homogenate; SF, starfish tissues; MOL-EXT, mollusc tissue extract; NT-EXT, newt extract; GB, gallbladder; AB, air bladder; GIT, gastrointestinal tract; DG, digestive gland; MG, midgut gland.

## Data Availability

Not applicable.
